# Impact of age on stage-specific mortality in patients with gastric cancer: A long-term prospective cohort study

**DOI:** 10.1371/journal.pone.0220660

**Published:** 2019-08-01

**Authors:** Jae Gon Lee, Shin Ah Kim, Chang Soo Eun, Dong Soo Han, Yong Sung Kim, Bo Youl Choi, Kyu Sang Song, Hyun Ja Kim, Chan Hyuk Park

**Affiliations:** 1 Department of Internal Medicine, Hanyang University Guri Hospital, Hanyang University College of Medicine, Guri, Korea; 2 Department of Food and Nutrition, Gangneung-Wonju National University, Gangneung, Korea; 3 Genome Editing Research Center, Korea Research Institute of Bioscience and Biotechnology (KRIBB), Daejeon, Korea; 4 Korea & Department of Functional Genomics, University of Science and Technology (UST), Daejeon, Korea; 5 Department of Preventive Medicine, Hanyang University College of Medicine, Seoul, Korea; 6 Department of Pathology, College of Medicine, Chungnam National University, Daejeon, Korea; National Cancer Center, JAPAN

## Abstract

Controversies exist regarding the impact of age on gastric cancer-related mortality according to cancer stage. In our prospective cohort study, we evaluated the impact of age on stage-specific mortality in patients with gastric cancer. Between 2002 and 2006, patients with newly diagnosed gastric cancer were recruited from two university-affiliated hospitals in Korea. Follow-up data were updated regularly based on medical records and telephone surveys. Patients were classified into four subgroups according to age: <50, 50–59, 60–69, and 70–79 years. A total of 448 patients were followed up for 81.6 months (interquartile range, 25.0–139.3 months). The number of patients with stage I, II, III, and IV disease was 247, 74, 88, and 39, respectively. Overall, age was an independent risk factor for gastric cancer-specific mortality (hazard ratio [HR], [95% confidence interval (CI)]: 1.53 [0.91–2.57], 1.88 [1.21–2.91], and 2.64 [1.69–4.14] in the 50–59, 60–69, and 70–79 years groups, respectively, with the <50 years group as reference). In patients with stage I and II gastric cancer, the 70–79 years group was associated with a significantly higher rate of cancer-specific mortality than the <50 years group (stage I: HR [95% CI], 9.55 [2.11–43.12]; stage II: HR [95% CI], 7.17 [2.32–22.18]). However, age was not an independently associated factor for cancer-specific mortality in patients with stage III and IV gastric cancer. Although age was an independent risk factor for gastric cancer-related mortality in patients with gastric cancer, its impact may differ depending on the stage of cancer.

## Introduction

Although the overall incidence and mortality rates of gastric cancer continue to decline, it remains the fifth most common cancer and the third leading cause of cancer-related death worldwide [1]. Operation, including surgical or endoscopic resection, is the only potentially curative treatment for gastric cancer. Patients with early-stage disease may have a high survival rate after surgery; however, patients with inoperable, recurrent, or metastatic disease receiving palliative chemotherapy or supportive care have a poor prognosis [2, 3].

Thus, surgical operability is closely related to the prognosis of patients with gastric cancer. Operability is determined not only by the stage of the tumor but also by the patient’s characteristics, including age [4]. Age is a non-modifiable risk factor for gastric cancer, and the incidence of gastric cancer increases with age [3]. Furthermore, the proportion of elderly patients with gastric cancer is increasing as the life expectancy of the general population increases in developed countries [5].

Elderly patients with gastric cancer are generally recognized as having a worse long-term prognosis than younger patients [6], but controversies exist regarding the impact of age on cancer-specific mortality [7]. Several studies have been conducted on the prognosis of young and elderly patients with gastric cancer; however, the results were inconclusive, and the reference age used varied across the studies [7–10]. In addition, there are no established treatment strategies for specific age groups of patients with gastric cancer [11]. In real-world practice, elderly patients are more likely to have conservative treatment without surgery because they are perceived as having a poorer prognosis after surgery than younger patients even in the same stage [7]. However, even if age is a clear prognostic factor in patients with gastric cancer, the influence of age on the prognosis of gastric cancer of different stages has not been well-established.

Therefore, a stage-specific approach is required to accurately analyze the impact of age on the mortality rate of gastric cancer. Such an approach may provide a basis for determining which therapeutic approaches should be applied to patients with gastric cancer according to age at different stages. Although a few studies on the impact of age by gastric cancer stage have been conducted [5, 12], there were limitations regarding accurate staging because population-based registry data were used in the studies. Thus, in this study, we aimed to evaluate the impact of age on stage-specific mortality in patients with gastric cancer using a hospital-based long-term cohort with reconstituted cancer stage data based on the latest American Joint Committee on Cancer (AJCC) staging system [13].

## Materials and methods

### Study cohort

In this cohort study, patients with gastric cancer were prospectively recruited from Chungnam University Hospital and Hanyang University Guri Hospital between March 2002 and September 2006. The inclusion criteria were as follows: (1) Patients who were diagnosed with gastric cancer through endoscopic biopsy and histopathologic examination, and (2) Patients who were aged 20–79. Gastric cancer was defined as tumors that were histologically diagnosed as gastric carcinoma according to the World Health Organization classification of tumors of the digestive system [14]. Patients with a lack of demographic information or cancer stage, or who were transferred to another hospital for surgical treatments were excluded from the cohort.

Demographic and clinicopathological data were collected at the time of enrollment. Written informed consent was obtained from all patients at the time of cohort enrollment. The study protocol conforms to ethical guidelines of the 1975 Declaration of Helsinki and was approved by the Institutional Review Board on Human Subjects Research and Ethics Committees Hanyang University Guri Hospital and the Chungnam National University Hospital Institutional Review Board.

### Measurements and definition

Demographic data, including age, sex, education level, smoking and drinking habits, and family history of gastric cancer, were collected through a structured interview. A family history of gastric cancer was defined as the disease in first-, second-, or third-degree relatives at any age. Participants who had smoked ≥400 cigarettes in their life and who currently smoke cigarettes were defined as “current smokers”. If a participant had smoked ≥400 cigarettes in his or her life but does not currently smoke, he or she was defined as a “former smoker”. Participants who had never smoked a cigarette, or who had smoked fewer than 400 cigarettes in their life were defined as “never smokers”. *Helicobacter pylori* infection status was determined by rapid urease test (CLO test, Kimberly-Clark, Ballard Medical Products, USA). Treatment modality was classified into the following three categories: operation, palliative chemotherapy, and supportive care alone. Both endoscopic resection and gastrectomy were regarded as operation.

Pathological data of all patients were reviewed in March 2018, and cancer stage was reconstituted according to the eighth edition of the AJCC tumor–node–metastasis (TNM) classification [13].

### Follow-up

Patients were followed up from the date of initial treatment for gastric cancer to the date of death or December 31, 2016. Medical records were reviewed to determine patient death and causes of death. For patients lost to follow-up, telephone surveys were conducted to identify survival and cause of death.

To assess the impact of age on the prognosis of patients with gastric cancer, patients were classified into four subgroups according to age: <50, 50–59, 60–69, and 70–79 years. Subsequently, overall and gastric cancer-specific survivals were evaluated according to the age subgroups.

### Statistical analyses

Demographic data were presented as numbers with proportions. Categorical variables among groups were compared using the chi-square or Fisher’s exact test. Survival analysis was performed using the Kaplan-Meier method with a log-rank test. Cox proportional hazard regression analysis was performed to assess risk factors for mortality. Significant variables (*P*-value <0.1) in the univariable analysis and potential confounding factors, including age, sex, and cancer stage were adjusted in the multivariable analysis. Risk of death was presented as hazard ratio (HR) with 95% confidence interval (CI). A *P*-value of <0.05 was considered to be statistically significant. Since our study included a relatively small sample size, we retrospectively calculated statistical power in the survival analyses to check comparability. All statistical analyses were performed using SAS version 9.4 (SAS Institute Inc., Cary, North Carolina, USA) and G*Power version 3.1.9.4 (Universität Düsseldorf, Germany) [15].

## Results

### Characteristics of patients according to age group

A total of 508 patients who were diagnosed with gastric cancer were included. Eighteen patients with a lack of demographic information and 42 patients who were transferred to another hospital before the treatment were excluded. As a result, 448 patients were finally included and followed up periodically in this study. A study flow diagram is shown in S1 Fig. Table 1 shows the baseline characteristics of patients according to age group. The mean age at diagnosis was 58.4 years (standard deviation, 11.9). Although most of the patients in the 50–59, 60–69, and 70–79 years groups were male (80.6%, 72.1%, and 74.1%, respectively), the proportion of female patients was relatively high in the <50 years group (male vs. female, 58.6% vs. 41.4%). A higher education status and a lower proportion of never smokers were identified in the <50 years group than in the other groups. The proportion of patients with a family history of gastric cancer did not differ among the groups (*P* = 0.213). In addition, *H*. *pylori* infection status and TNM stage did not differ among the groups (*P* = 0.352 and *P* = 0.298, respectively).

**Table 1:** Baseline characteristics of patients according to age group.

Variable	Age group, year				*P* value	Total (n = 448)
	<50 (n = 111)	50–59 (n = 98)	60–69 (n = 154)	70–79 (n = 85)		
Sex, n (%)					0.004	
Male	65 (58.6)	79 (80.6)	111 (72.1)	63 (74.1)		318
Female	46 (41.4)	19 (19.4)	43 (27.9)	22 (25.9)		130
Registered hospital, n (%)						
Chungnam University Hospital	72 (64.9)	65 (66.3)	83 (53.9)	38 (44.7)		258
Hanyang University Guri Hospital	39 (35.1)	33 (33.7)	71 (46.1)	47 (55.3)		190
Education, n (%)					<0.001	
College or higher	27 (24.3)	10 (10.2)	11 (7.1)	3 (3.5)		51
Middle or high school	68 (61.3)	62 (63.3)	65 (42.2)	17 (20.0)		212
Elementary school or none	12 (10.8)	22 (22.4)	66 (42.9)	53 (62.4)		153
Unknown	4 (3.6)	4 (4.1)	12 (7.8)	12 (14.1)		32
Smoking habit, n (%)					<0.001	
Never smoker	49 (44.1)	22 (22.4)	50 (32.5)	26 (30.6)		147
Former smoker	24 (21.6)	25 (25.5)	59 (38.3)	33 (38.8)		141
Current smoker	38 (34.2)	51 (52.0)	45 (29.2)	25 (29.4)		159
Unknown	0 (0.0)	0 (0.0)	0 (0.0)	1 (1.2)		1
Alcohol consumption, n (%)					0.090	
Never drinker	33 (29.7)	21 (21.4)	58 (37.7)	27 (31.8)		139
Former drinker	15 (13.5)	22 (22.4)	29 (18.8)	18 (21.2)		84
Current drinker	63 (56.8)	55 (56.1)	67 (43.5)	40 (47.1)		225
Family history of gastric cancer, n (%)					0.213	
Absence	79 (71.2)	79 (80.6)	122 (79.2)	70 (82.4)		350
Presence	32 (28.8)	19 (19.4)	32 (20.8)	15 (17.6)		98
*Helicobacter pylori* infection, n (%)					0.352	
Absence	29 (26.1)	29 (29.6)	61 (39.6)	34 (40.0)		153
Presence	34 (30.6)	28 (28.6)	44 (28.6)	23 (27.1)		129
Unknown	48 (43.2)	41 (41.8)	49 (31.8)	28 (32.9)		166
TNM stage, n (%)					0.298	
I	55 (49.5)	63 (64.3)	89 (57.8)	40 (47.1)		247
II	19 (17.1)	11 (11.2)	27 (17.5)	17 (20.0)		74
III	26 (23.4)	19 (19.4)	26 (16.9)	17 (20.0)		88
IV	11 (9.9)	5 (5.1)	12 (7.8)	11 (12.9)		39
Treatment for gastric cancer, n (%)					0.074	
Operation	100 (90.1)	94 (95.9)	143 (92.9)	73 (85.9)		410
Palliative chemotherapy	5 (4.5)	1 (1.0)	2 (1.3)	1 (1.2)		9
Supportive care alone	6 (5.4)	3 (3.1)	9 (5.8)	11 (12.9)		29

TNM, tumor-node-metastasis

### Overall and gastric cancer-specific survivals

**Fig 1:**
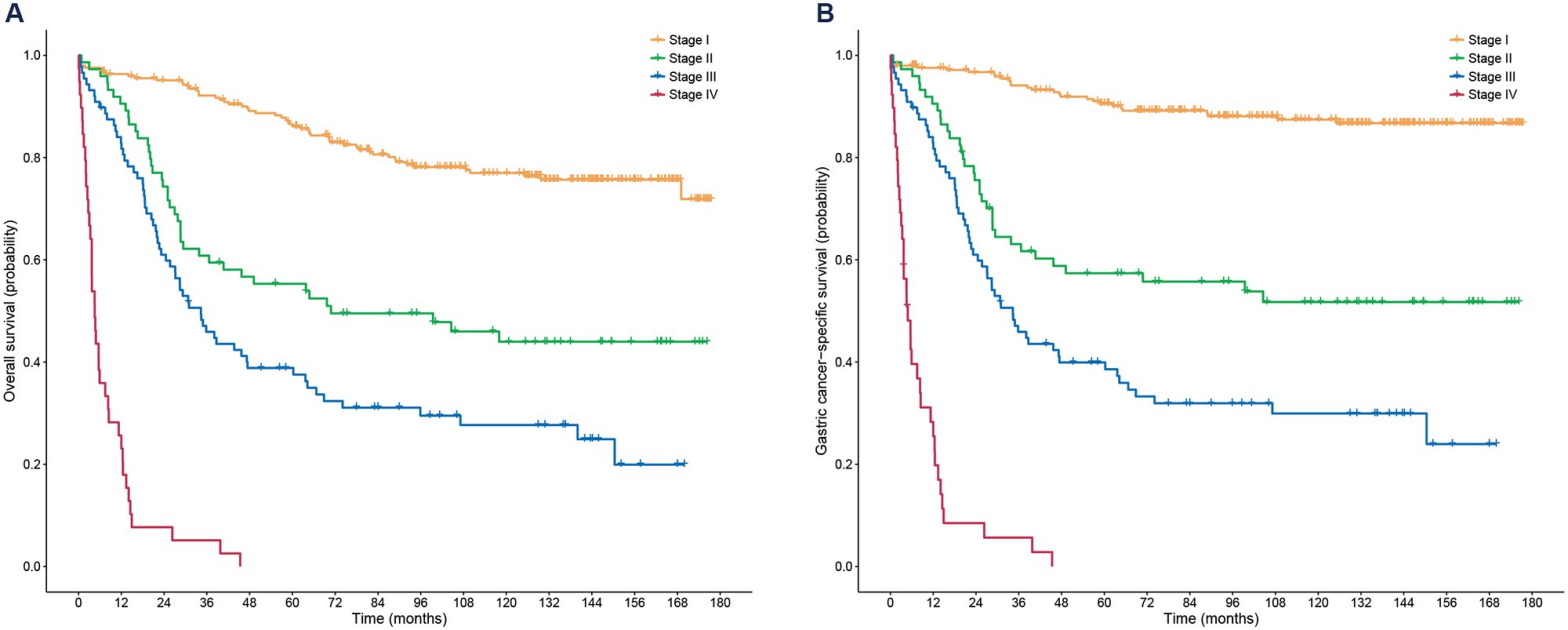
Kaplan-Meier plots for overall (A) and gastric cancer-specific survival (B) according to the eighth edition of the AJCC TNM staging system. AJCC, American Joint Committee on Cancer; TNM, tumor-node-metastasis.

Fig 1 shows the Kaplan-Meier plots for overall and gastric cancer-specific survivals. Overall median follow-up duration was 81.6 months (interquartile range [IQR], 25.0–139.3). The five-year overall survival rates of patients with stage I, II, III, and IV were 86.1% (95% CI, 81.8–90.6%), 55.3% (95% CI, 45.1–67.9%), 38.8% (95% CI, 29.8–50.6%), and 0%, respectively. In addition, the five-year gastric cancer-specific survival rates were 90.6% (95% CI, 86.9–94.4%), 57.4% (95% CI, 47.0–70.0%), 39.9% (95% CI, 30.8–51.7%), and 0%, respectively.

As shown in the Cox-proportional hazard model in Tables 2 and 3, both age and cancer stage were independent risk factors for mortality. Compared to patients aged <50 years, those aged 70–79 years had a 3.2-times higher risk of overall mortality and a 2.6-times higher risk of gastric cancer-specific mortality in the multivariable analysis. In the retrospective statistical power calculations, the statistical power for overall survival was 36.5% (<50 vs. 50–59 years), 97.1% (<50 vs. 60–69 years), and 99.9% (<50 vs. 70–79 years), respectively.

**Table 2:** Cox proportional hazard models for overall mortality.

Variable	Univariable analysis		Multivariable analysis	
	HR (95% CI)	*P* value	HR (95% CI)	*P* value
Age group, year				
<50	1.000		1.000	
50–59	0.970 (0.600–1.568)	0.902	1.483 (0.911–2.413)	0.113
60–69	1.527 (1.022–2.280)	0.039	2.124 (1.417–3.186)	<0.001
70–79	2.974 (1.963–4.506)	<0.001	3.167 (2.088–4.801)	<0.001
Sex				
Female	1.000		1.000	
Male	1.039 (0.758–1.425)	0.810	1.200 (0.833–1.730)	0.328
Education				
Elementary school or none	1.000		1.000	
Middle or high school	0.722 (0.530–0.983)	0.038	1.145 (0.813–1.613)	0.438
College or higher	0.616 (0.369–1.029)	0.064	1.204 (0.675–2.148)	0.529
Unknown	1.354 (0.800–2.291)	0.259	1.705 (0.998–2.911)	0.051
Smoking				
Never smoker	1.000			
Former smoker	1.098 (0.766–1.573)	0.612		
Current smoker	1.284 (0.912–1.808)	0.152		
Unknown	0.000 (0.000–0.000)	0.959		
Alcohol consumption				
Never drinker	1.000		1.000	
Former or current drinker	0.800 (0.595–1.076)	0.139	0.783 (0.559–1.096)	0.154
Family history of gastric cancer				
Absence	1.000		1.000	
Presence	0.581 (0.394–0.858)	0.006	0.767 (0.513–1.145)	0.194
*Helicobacter pylori* infection				
Absence	1.000			
Presence	1.163 (0.825–1.641)	0.389		
Unknown	0.816 (0.570–1.169)	0.268		
TNM stage				
I	1.000		1.000	
II	3.209 (2.134–4.827)	<0.001	3.315 (2.200–4.996)	<0.001
III	5.505 (3.818–7.939)	<0.001	5.722 (3.960–8.266)	<0.001
IV	36.474 (22.875–58.158)	<0.001	36.860 (22.853–59.452)	<0.001

HR, hazard ratio; CI, confidence interval; TNM, tumor-node-metastasis

**Table 3:** Cox proportional hazard models for gastric cancer-specific mortality.

Variable	Univariable analysis		Multivariable analysis	
	HR (95% CI)	*P* value	HR (95% CI)	*P* value
Age group, year				
<50	1.000		1.000	
50–59	0.923 (0.555–1.534)	0.756	1.531 (0.913–2.566)	0.106
60–69	1.260 (0.817–1.943)	0.296	1.876 (1.211–2.907)	0.005
70–79	2.438 (1.558–3.815)	<0.001	2.642 (1.687–4.138)	<0.001
Sex				
Female	1.000		1.000	
Male	0.870 (0.621–1.217)	0.416	1.056 (0.712–1.567)	0.786
Education				
Elementary school or none	1.000		1.000	
Middle or high school	0.779 (0.552–1.098)	0.154	1.254 (0.852–1.846)	0.251
College or higher	0.652 (0.370–1.149)	0.139	1.264 (0.665–2.400)	0.475
Unknown	1.405 (0.785–2.515)	0.252	1.819 (1.003–3.300)	0.049
Smoking				
Never smoker	1.000			
Former smoker	0.961 (0.647–1.428)	0.844		
Current smoker	1.143 (0.787–1.659)	0.483		
Unknown	0.000 (0.000–0.000)	0.963		
Alcohol consumption				
Never drinker	1.000		1.000	
Former or current drinker	0.697 (0.506–0.961)	0.028	0.721 (0.498–1.042)	0.082
Family history of gastric cancer				
Absence	1.000		1.000	
Presence	0.608 (0.397–0.931)	0.022	0.827 (0.531–1.289)	0.402
*Helicobacter pylori* infection				
Absence	1.000			
Presence	1.162 (0.794–1.699)	0.440		
Unknown	0.796 (0.534–1.186)	0.262		
TNM stage				
I	1.000		1.000	
II	5.004 (3.046–8.221)	<0.001	5.163 (3.137–8.497)	<0.001
III	9.252 (5.917–14.466)	<0.001	9.621 (6.141–15.074)	<0.001
IV	55.393 (32.461–94.525)	<0.001	57.340 (33.139–99.217)	<0.001

HR, hazard ratio; CI, confidence interval; TNM, tumor-node-metastasis

### Survival outcomes according to age group

**Fig 2:**
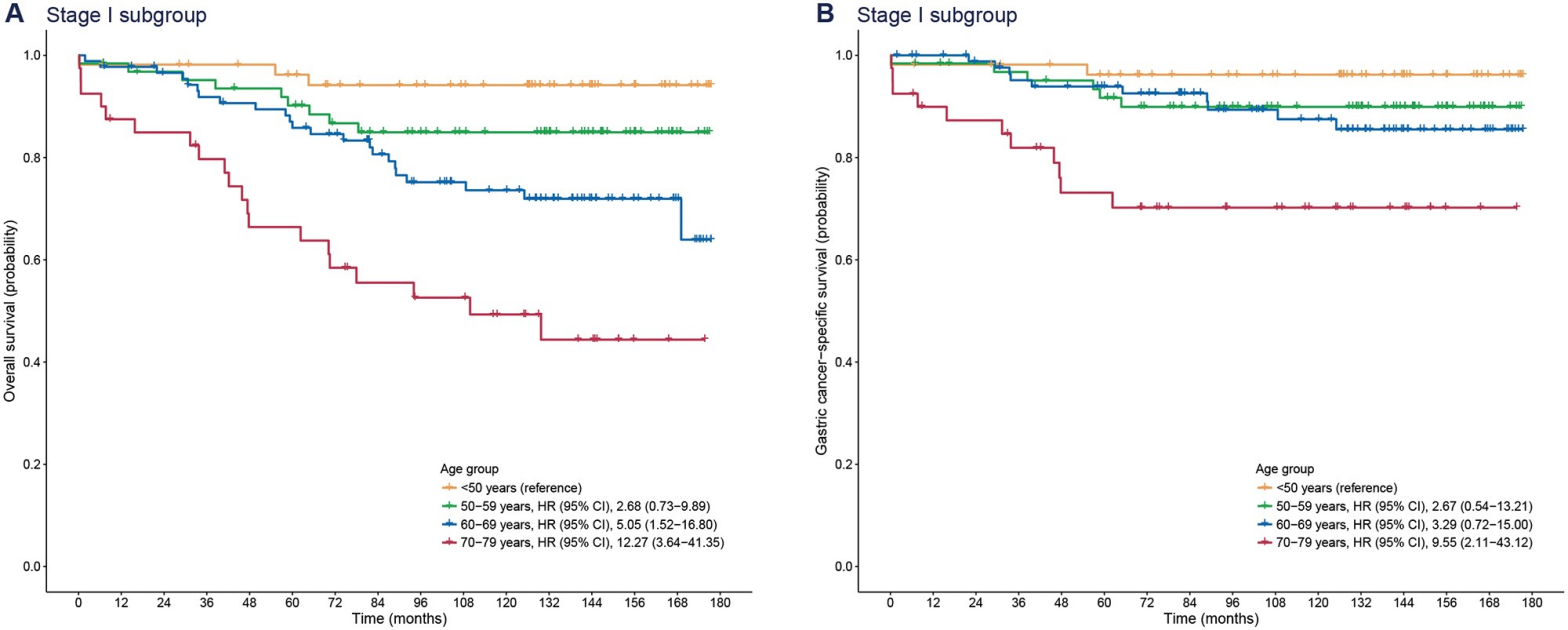
Kaplan-Meier plots for overall (A) and gastric cancer-specific survival (B) according to age group in patients with stage I gastric cancer. HR, hazard ratio; CI, confidence interval.

Over a median follow-up period of 125.9 months (IQR, 70.3–146.5), there were 29 deaths from gastric cancer out of 247 patients with stage I gastric cancer. Compared with patients aged <50 years, those aged 70–79 years had a significantly higher rate of gastric cancer-specific mortality (HR [95% CI], 9.55 [2.11–43.12]), whereas the 50–59 and 60–69 years groups were not associated with an increased gastric cancer-specific mortality (50–59 years: HR [95% CI], 2.67 [0.54–13.21]; 60–69 years: HR [95% CI], 3.29 [0.72–15.0]) (Fig 2). In terms of overall survival, patients aged 60–69 and 70–79 years were inferior to those aged <50 years due to mortality from causes other than gastric cancer (60–69 years: HR [95% CI], 5.05 [1.52–16.80]; 70–79 years: HR [95% CI], 12.27 [3.64–41.35]). The impact of age on overall and gastric cancer-specific mortality was greater in patients with stage I gastric cancer than in the entire study population.

**Fig 3:**
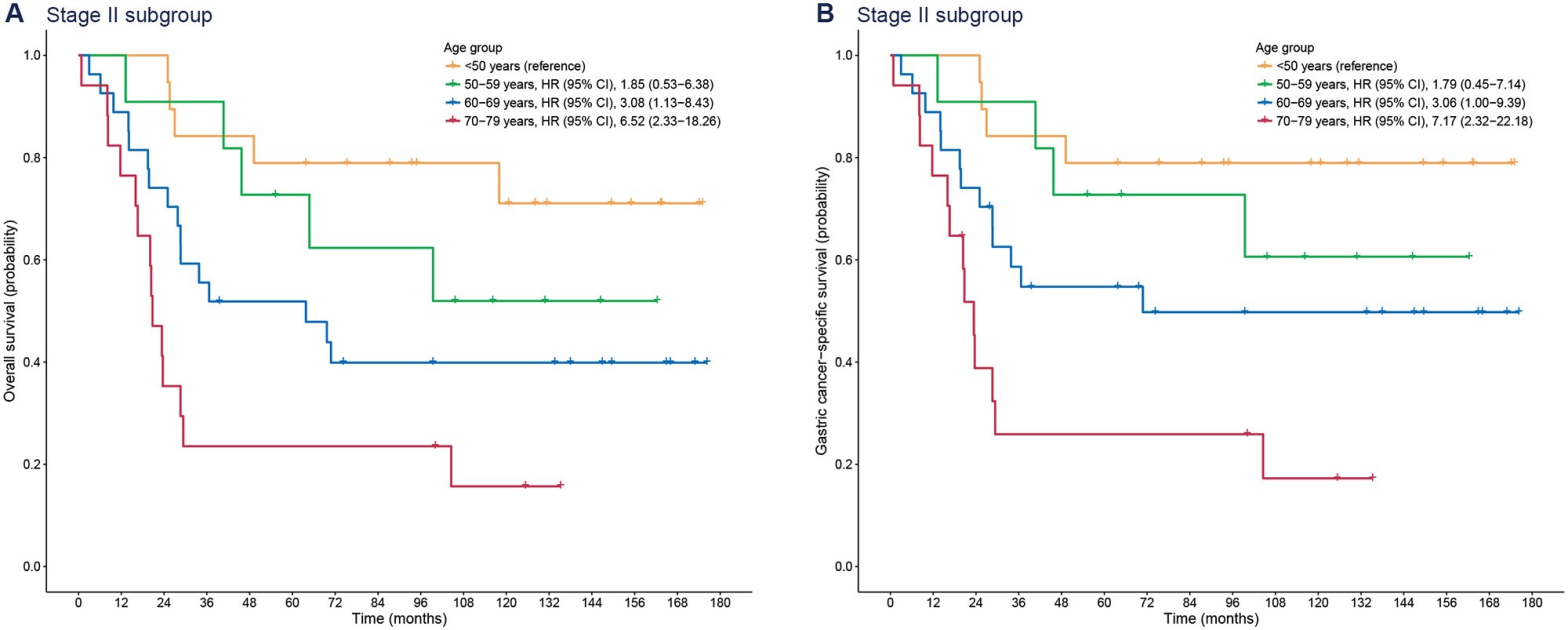
Kaplan-Meier plots for overall (A) and gastric cancer-specific survival (B) according to age group in patients with stage II gastric cancer. HR, hazard ratio; CI, confidence interval.

Fig 3 shows survival curves in patients with stage II gastric cancer. Although the HRs of age of this subgroup decreased compared to the HRs of the stage I cancer subgroup, old age (70–79 years) was an independent risk factor for overall and gastric cancer-specific mortality (overall mortality: HR [95% CI], 6.52 [2.33–18.26]; gastric cancer-specific mortality: HR [95% CI], 7.17 [2.32–22.18]).

**Fig 4:**
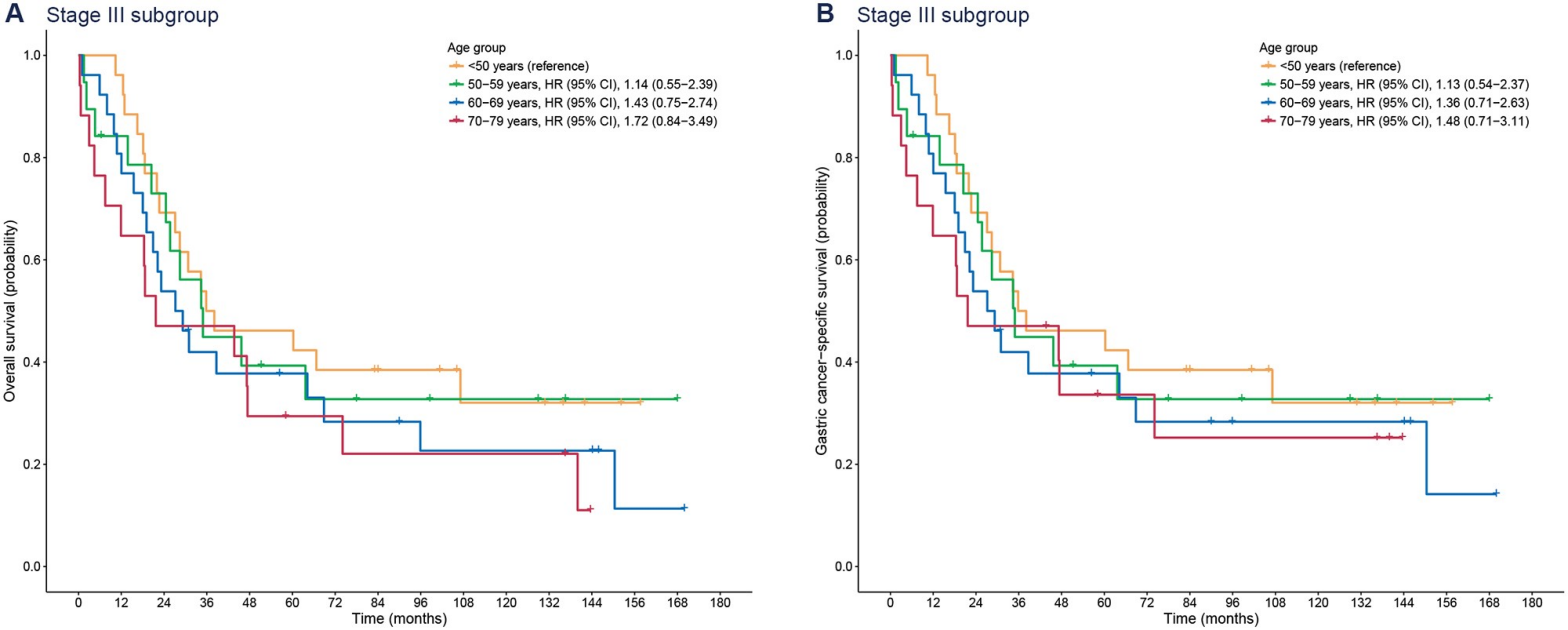
Kaplan-Meier plots for overall (A) and gastric cancer-specific survival (B) according to age group in patients with stage III gastric cancer. HR, hazard ratio; CI, confidence interval.

In contrast to the results of stage I and II subgroups, the impact of age on survival was not identified in the stage III subgroup, as shown in Fig 4 (*P* = 0.445 for overall survival and *P* = 0.697 for gastric cancer-specific survival). In the subgroup of patients with stage III gastric cancer, only three patients (3.4%) died from causes other than gastric cancer (two were aged 70–79 years, and the other was aged 60–69 years). Moreover, within five years, only one patient (1.1%) died from a cause other than gastric cancer. In other words, five-year survivals of patients mostly depended on gastric cancer itself rather than on the age of the patients with advanced-stage cancer.

**Fig 5:**
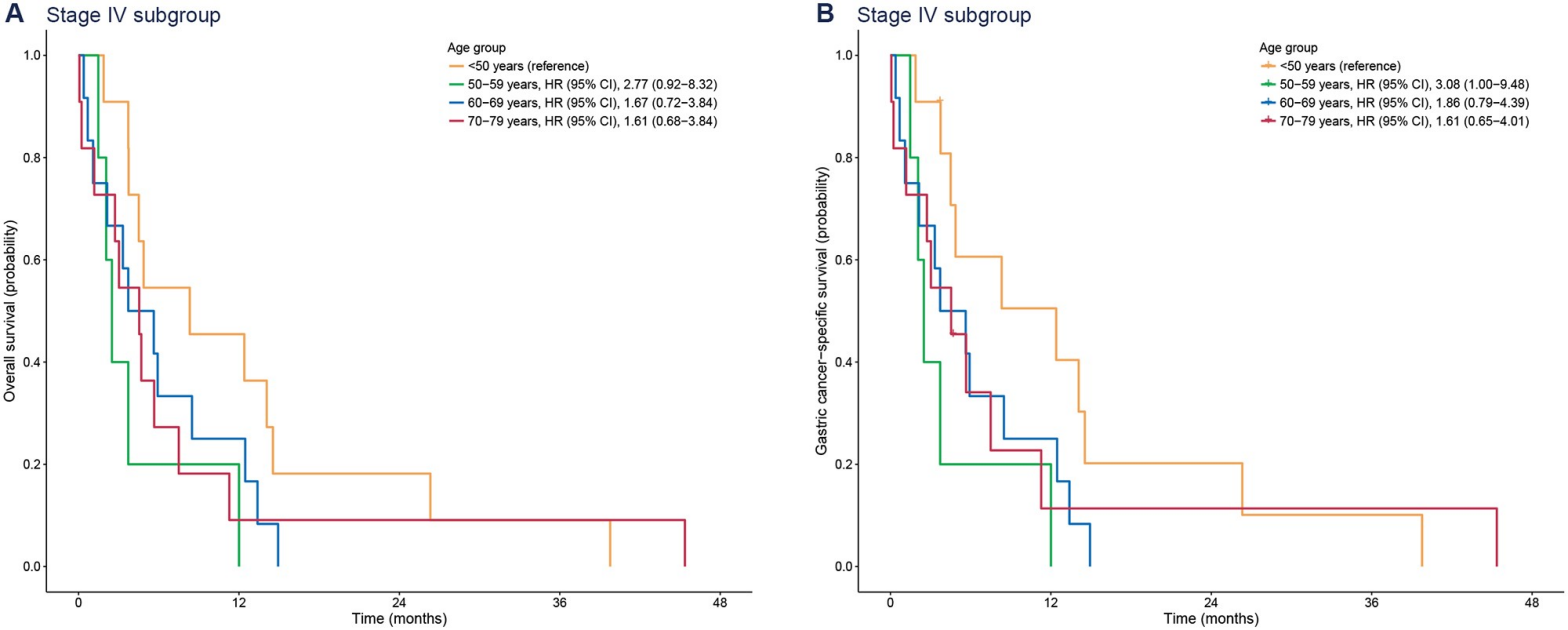
Kaplan-Meier plots for overall (A) and gastric cancer-specific survival (B) according to age group in patients with stage IV gastric cancer. HR, hazard ratio; CI, confidence interval.

In the subgroup of patients with stage IV gastric cancer, either palliative chemotherapy or supportive care was performed. Similar to the results of the stage III subgroup, age was not associated with survival (*P* = 0.291 for overall survival and *P* = 0.215 for gastric cancer-specific survival) (Fig 5).

### Early postoperative mortality according to age group

Additionally, we analyzed 30-day postoperative mortality to assess the impact of age on perioperative mortality. Among 410 patients who underwent an operation, eight died within 30 days of operation. The 30-day mortality was highest in the 70–79 years group among the four age groups (<50 vs. 50–59 vs. 60–69 vs. 70–79 years: 1.0% vs. 1.1% vs. 0.7 vs. 6.8%, *P* = 0.023). Of five patients who died within 30 days after operation in the 70–79 years group, three, one, and one had stage I, II, and III gastric cancer, respectively. In other words, 30-day postoperative mortality was 7.5%, 5.9%, and 5.9% in elderly patients with stage I, II, and III gastric cancer, respectively (*P*>0.999).

## Discussion

In this hospital-based, long-term cohort study, we investigated the impact of age on stage-specific mortality in patients with gastric cancer, with the reconstituted stage based on the eighth edition of the AJCC staging system. Overall, higher age at diagnosis was correlated with a worse gastric cancer-specific prognosis. Specifically, the mortality rate of gastric cancer increased with age in patients with stage I or II gastric cancer whereas the mortality rate of gastric cancer was not affected by an increase in age in patients with stage III or IV gastric cancer.

The incidence of gastric cancer increases with age [3]. Aging is associated with increased susceptibility to injury, delayed healing of the gastric mucosa, and increased expression of cancer stem cell markers [16, 17]. Additionally, *H*. *pylori*-associated gastritis increases with age [18]. However, it had been unclear whether the prognosis of already established gastric cancer worsens with age.

As expected, overall mortality in patients with stage I and II gastric cancer was higher in the elderly groups than in the younger groups. This is likely because mortality from any cause other than gastric cancer is higher in elderly patients than in younger patients. In our study, we showed that gastric cancer-specific mortality was also higher in patients aged 70–79 years than in those aged <50 years with stage I and II gastric cancers. This finding is consistent with a previous population-based cohort study [12]. Relatively high gastric cancer-specific mortality in elderly patients is presumed to be associated with inadequate treatment of these patients. The primary curative treatment for gastric cancer is surgical resection, and adjunctive chemotherapy or radiotherapy may also be required [19]. Gastrectomy with extended (D2) lymphadenectomy is currently the standard surgical procedure for patients with resectable gastric cancer, with a goal of examining at least ≥15 lymph nodes [19, 20]. The total lymph node number has been reported to play an important role in prognostic evaluation and treatment decisions [12, 20]. However, elderly patients with gastric cancer have been reported to be associated with partial gastrectomy and inadequate lymph node harvesting, which may result in insufficient treatment and inadequate staging [4, 21]. Inadequate staging may be associated with inadequate postoperative treatment, including adjuvant chemotherapy or radiotherapy. Moreover, elderly patients have been reported to receive adjuvant chemotherapy less frequently and have lower completion rates due to poor performance status [4].

In contrast to the results of patients with stage I or II gastric cancer, there was no difference in mortality according to age group in patients with stage III or IV gastric cancer. This finding implies that the impact of cancer stage outweighs the impact of age on mortality in advanced-stage diseases. Young patients with gastric cancer, usually defined as patients aged <40 years, have been reported to have a higher incidence of undifferentiated-type carcinoma and metastatic disease [9]. However, the prognosis of young patients with gastric cancer has been controversial [22–24], and young patients with early-stage gastric cancer who could undergo curative resection reportedly had a good prognosis [23, 25]. These findings suggest that cancer stage and disease behavior are more important in the prognosis of gastric cancer than age itself, especially in advanced-stage disease. In clinical practice, many physicians are reluctant to perform surgery on elderly patients with stage III gastric cancer. However, the results of this study suggest that age itself is not an independent prognostic factor in stage III gastric cancer patients. Therefore, it may be inappropriate to avoid surgery because of the increased age alone. It may be necessary to consider a patients’ performance status and/or comorbidities rather than age alone when deciding whether to perform surgery.

However, relatively higher perioperative mortality may be a concern in the surgical treatment of elderly patients. In our study, 6.8% of patients aged 70–79 years who underwent operation died within 30 days after the operation. Previous studies have also reported that elderly patients are more likely to have comorbidities and poor performance status, and the surgical risk increases with age [5, 11]. Patients aged >70 years were reported to have a higher chance of developing postoperative complications [4]. Moreover, the occurrence of postoperative complications, which can lead to prolonged inflammation, was reported to have a negative effect on the long-term prognosis of patients with gastric cancer even if the tumor is resected completely [26]. In addition, a previous study reported that increased age and poor performance status were risk factors for anastomotic leakage after gastrectomy, and the occurrence of anastomotic leakage was a major independent predictor for long-term mortality in patients with gastric cancer [27]. Based on the results of our study, however, perioperative mortality in elderly patients appeared to be independent of cancer stage. Although perioperative mortality was higher in elderly patients than in younger patients, it did not differ across cancer stage in elderly patients. In other words, the risk of perioperative mortality may be an issue in elderly patients regardless of cancer stage.

Although our study demonstrated that the impact of age on prognosis depended on cancer stage in patients with gastric cancer, it has several limitations. First, exact data for patients’ performance status were not collected at the time of establishing the cohort. Although treatment methods did not differ significantly among the age groups, the proportion of patients who underwent supportive care alone tended to be higher in the 70–79 years group. Various factors, including performance status, might have affected the selection of treatment modalities. Second, a relatively small number of patients were included compared to other population-based studies. The statistical power of our study was relatively low due to the small sample size and low effect size in the comparison between <50 vs. 50–59 years groups. Although statistical power was sufficiently high between <50 vs. 60–69 years groups and <50 vs. 70–79 years groups, lack of significance in the findings between <50 vs. 50–59 years groups, especially in the subgroup analyses, should be cautiously interpreted. Third, in some patients, the cause of death was determined based on a telephone survey rather than from secured medical records, possibly leading to inaccuracies in some cases.

Despite these limitations, our data provide a better understanding of the impact of age on the prognosis of gastric cancer. This was the first long-term cohort study that evaluated the impact of age on stage-specific mortality with the reconstituted stage based on the eighth edition of the AJCC staging system. In conclusion, the overall and gastric cancer-specific mortalities increased with age in patients with stage I or II gastric cancer. However, overall and gastric cancer-specific mortalities were not affected by age in patients with stage III or IV gastric cancer.

## Supporting information

S1 Fig Study flow diagram. (TIF)
