# Supplementary figures and images for: Impact of age on stage-specific mortality in patients with gastric cancer: A long-term prospective cohort study
Source: PLoS One. 2019 Aug 1;14(8):e0220660. doi: 10.1371/journal.pone.0220660 (PMC6675285; doi:10.1371/journal.pone.0220660)

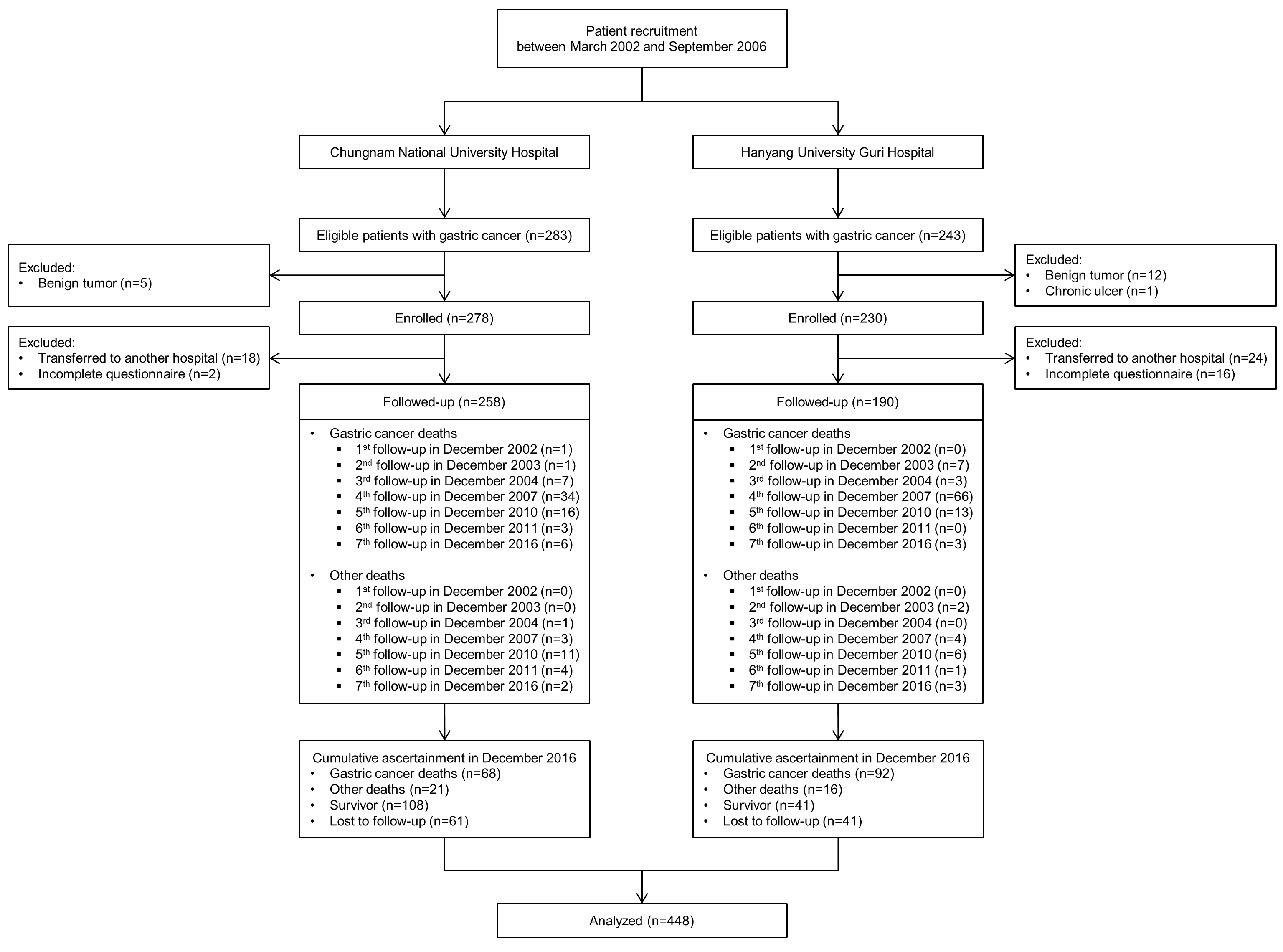

Supplement: S1 Fig — (TIF) [file pone.0220660.s001.tif]
